# Ecological Structuring of Temperate Bacteriophages in the Inflammatory Bowel Disease-Affected Gut

**DOI:** 10.3390/microorganisms8111663

**Published:** 2020-10-27

**Authors:** Hiroki Nishiyama, Hisashi Endo, Romain Blanc-Mathieu, Hiroyuki Ogata

**Affiliations:** 1 Bioinformatics Center, Institute for Chemical Research, Kyoto University, Uji 611-0011, Japan; hiroki@kuicr.kyoto-u.ac.jp (H.N.); endo@scl.kyoto-u.ac.jp (H.E.); 2 Laboratoire de Physiologie Cellulaire & Végétale, CEA, CNRS, INRA, IRIG, Université Grenoble Alpes, 38000 Grenoble, France; romain.blancmathieu@cea.fr

**Keywords:** temperate bacteriophage, inflammatory bowel disease, ulcerative colitis, Crohn’s disease, gut microbiota

## Abstract

The aim of this study was to elucidate the ecological structure of the human gut temperate bacteriophage community and its role in inflammatory bowel disease (IBD). Temperate bacteriophages make up a large proportion of the human gut microbiota and are likely to play a role in IBD pathogenesis. However, many of these bacteriophages await characterization in reference databases. Therefore, we conducted a large-scale reconstruction of temperate bacteriophage and bacterial genomes from the whole-metagenome sequence data generated by the IBD Multi’omics Database project. By associating phages with their hosts via genome comparisons, we found that temperate bacteriophages infect a phylogenetically wide range of bacteria. The majority of variance in bacteriophage community composition was explained by variation among individuals, but differences in the abundance of temperate bacteriophages were identified between IBD and non-IBD patients. Of note, in active ulcerative colitis patients, temperate bacteriophages infecting *Bacteroides uniformis* and *Bacteroides thetaiotaomicron*—two species experimentally proven to be beneficial to gut homeostasis—were over-represented, whereas their hosts were under-represented in comparison with non-IBD patients. Supporting the mounting evidence that gut viral community plays a vital role in IBD, our results show potential association between temperate bacteriophages and IBD pathogenesis.

## 1. Introduction

Inflammatory bowel disease (IBD) encompasses a group of intestinal disorders, the most common of which are Crohn’s disease (CD) and ulcerative colitis (UC). These disorders are characterized by chronic inflammation that occurs throughout multiple layers of the gastrointestinal tract in CD and in the inner layer of the colon in UC. The exact cause of IBD is not clear; however, the gut microbiota, together with host genetics and environmental factors (e.g., diet, antibiotics, and drugs), plays an important role in the pathogenesis of IBD [1,2]. Although bacteria have been the focus of most IBD-related gut microbiota studies, recent studies have shown that bacteriophages in the gut may also play an important role in the pathogenesis of IBD [3,4,5].

The human gut bacteriophage community is thought to be largely composed of temperate bacteriophages [6], which are characterized by their ability to replicate through two different life cycles: the lysogenic cycle and the lytic cycle [7]. In the lysogenic cycle, temperate bacteriophages are integrated into their host genome in the form of prophages. During this process, viral genes, including those associated with anti-microbial activity, virulence, and toxin production, are horizontally transferred to the host. Upon induction, temperate bacteriophages enter the lytic cycle, in which virions are produced from prophages and released into the outer environment following lysis of the host bacterium.

Recent studies on gut bacteriophage communities in IBD-affected individuals suggest that *Caudovirales* are associated with the pathogenesis of IBD [3,4]. However, insights based on viral taxonomy require careful consideration because the majority of viruses in the human gut are yet to be taxonomically classified [8]. Furthermore, viral taxonomy is under constant review by the International Committee on the Taxonomy of Viruses (ICTV) to accommodate newly sequenced viral genomes [9]. For example, the definition of the *Caudovirales* order was recently changed to include two new families: *Ackermannviridae* and *Herelleviridae* [6]. To address these problems, we aimed to characterize the ecological structure of functional temperate bacteriophage communities in the guts of IBD patients by identifying associations between these viruses and their bacterial hosts via reference-independent methods using a large publicly available microbiome dataset.

## 2. Materials and Methods

### 2.1. IBDMDB Data Depository

This study was conducted using publicly available whole-metagenome shotgun sequencing (WMGS) data generated by the IBD Multi’omics Database (IBDMDB) research team as part of the Human Microbiome Project 2 [1,10]. Specifically, 1338 sets of quality-filtered WMGS data were used. Sequences (i.e., reads) were generated via DNA sequencing using the Illumina HiSeq (2 × 101 bp) system (Illumina, San Diego, CA, USA) from longitudinal stool samples from 65 CD patients, 38 UC patients, and 27 non-IBD patients. Non-IBD patients had gastrointestinal symptoms but were not diagnosed with IBD [1]. Quality control of WMGS data was performed by the IBDMDB research team [1]. Briefly, the AnADAMA2 pipeline (http://huttenhower.sph.harvard.edu/anadama2) was used to remove low quality regions from reads, and any reads matching the human genome were discarded. All sequence data, including the WMGS metadata, were downloaded from the IBDMDB data depository (http://ibdmdb.org).

### 2.2. Assessment of Disease Activity Among CD and UC Patients

CD patients with a Harvey–Bradshaw index (HBI) score ≥5 were identified as having active CD [11]. CD patients with an HBI score <5 were classified as having inactive CD. Among UC patients, those with a simple clinical colitis activity index (SCCAI) score ≥5 were identified as having active UC [12], whereas those with a SCCAI score <5 were identified as having inactive UC.

### 2.3. De Novo Contig Assembly

Quality-filtered WMGS data from the 1327 stool samples with sufficient sequencing depth (i.e., minimum of 10,000 reads) were selected for *de novo* assembly. Reads from each stool sample were assembled into contiguous sequences (i.e., contigs) using MEGAHIT (version 1.1.4) (--kmin 21, --kmax 251, --k-step 10) [13]. To assemble the genomes from the microbes present at low abundance, we also co-assembled the reads from the stool samples taken from the same individual using the same method.

### 2.4. Generation of Viral Operational Taxonomic Units (OTUs) for Temperate Bacteriophages

Viral regions within contigs were predicted using VirSorter (version 1.0.5) (--db 2, --diamond) [14]. To reduce false identification, predicted viral regions with at least one hallmark viral gene and/or enrichment of either viral-like genes or non-*Caudovirales* viral genes (i.e., VirSorter categories 1, 2, 4, and 5) were selected for further analyses. The selected viral regions were grouped into viral OTUs at 95% sequence similarity using CD-HIT-EST (version 4.6.8) (-c 0.95, -aS 0.85, -n 10, -g 1) [15]. To identify viral OTUs representing temperate bacteriophages, MetaGeneMark (version 4.30) was first used to derive translated peptide sequences from the viral regions [16]. Subsequently, the peptide sequences were searched against the Pfam-A database (version 33.0) using hmmscan (version 3.3) (--cut_ga) [17,18]. Viral OTUs encoding one or more of the following viral integrase protein domains were defined as temperate bacteriophage and were selected for the subsequent analyses: PF00589, PF02899, PF09003, PF14659, PF13356, PF13495, PF12834, PF12835, PF13009, PF14657, PF02914, PF02316, PF02920, PF09299. Taxonomic classification of the viral OTUs was performed by classifying representative sequences (the longest) from each OTU using vConTACT v2.0 (version 0.9.12) (--rel-mode Diamond, --db ProkaryoticViralRefSeq94-Merged) [19].

### 2.5. Reconstruction of Bacterial Genomes via Metagenomic Binning

Contigs were binned into metagenome-assembled genomes (MAGs) using METAWRAP (version 1.1.8) (binning, --metabat2, --maxbin2, --concoct) [20]. Briefly, reads from the same individual were mapped on the contigs using Burrows–Wheeler aligner [21]. Contigs ≥1000 bp in length (1500 bp for MetaBAT2) were then binned into MAGs based on their read coverage using three binning tools: MaxBin2 (version 2.2.5), MetaBAT2 (version 2.12.1), and CONCOCT (version 1.0.0) [22,23,24]. Subsequently, METAWRAP binning_refiner was used to hybridize MAGs generated via different binning tools and the best set of MAGs was chosen based on completion and contamination statistics calculated by CheckM (version 1.0.13) (lineage_wf) [20,25]. Of these, MAGs with a minimum completion of 70% and maximum contamination of 10% were dereplicated (i.e., grouped) into MAG clusters with a minimum genome-wide average nucleotide identity (gANI) threshold of 99% and a minimum aligned fraction threshold of 60% using dRep (version 2.2.2) [26]. Representative MAGs were chosen for each cluster based on completeness, contamination, strain heterogeneity, N50, and size [26]. NCBI taxonomy was determined for each representative MAG by placing them into a protein reference tree using GTDB-Tk (version 1.0.2) (classify_wf) [27]. Six MAG clusters assigned to archaea were removed. The resulting collection of 3133 bacterial MAG clusters were selected for subsequent analyses.

### 2.6. Construction of a Bacterial Phylogenetic Tree

A bacterial phylogenetic tree was constructed by first extracting translated peptide sequences from the representative draft genome of each MAG cluster using MetaGeneMark (version 4.30) [16]. The translated peptide sequences were then used to generate a phylogenetic tree using PhyloPhlAn (version 0.99) (--user_tree) [28]. Briefly, input peptides sequences matching to PhyloPhlAn’s collection of 400 marker proteins (i.e., proteins broadly conserved in bacteria and archaea) were identified using USEARCH (version 5.2.236) [29]. Subsequently, these proteins are aligned with MUSCLE (version 3.8.31) [30] and reconstructed into a phylogenetic tree with FastTree (version 2.1.11) [31]. The visual representation of the phylogenetic tree was generated using iTOL (version 5.6) [32].

### 2.7. Assignment of Hosts to Viral OTUs

Two strategies based on previous studies were used for host assignment of viral OTUs: binning-and basic local alignment search tool (BLAST)-based methods [33,34,35]. In the binning-based method, a MAG cluster was assigned as the host of a viral OTU if the contigs from the MAG included a region corresponding to the viral OTU. In the BLAST-based method, bacterial sequences adjacent to prophage sequences (i.e., VirSorter categories 4 and 5) were aligned against MAGs using BLASTn in the BLAST+ package (version 2.8.1) (-perc_identity 95, -evalue 1e-50, -word_size 11) [36]. If both the upstream and downstream regions of a prophage sequence in a viral OTU aligned with contigs from a MAG cluster with sequence similarity >95% and query coverage >80%, the MAG cluster was considered to be the host. Long queries were trimmed to 1000 bp starting from the edge of the selected prophage region. Prophage sequences that were not flanked on both sides by sequences ≥250 bp were excluded from the host assignment process.

### 2.8. Mapping Reads to Viral OTUs and MAGs

Quality-filtered reads from 1338 gut metagenomes were mapped to the representative sequences of each viral OTU and MAG cluster using bowtie2 (version 2.3.4.3) (--maxins 1000) [37]. Resulting bam files were processed using CoverM (version 0.3.0, https://github.com/wwood/CoverM/) (--proper-pairs-only) so that only reads that aligned across ≥80% of their length with a minimum of 95% sequence identity were retained. The bam files were then further processed using samtools (version 1.9) (-f 3, -F 2304) [38], and only the primary alignments from the properly paired reads were retained. Counts of mapped read pairs were retrieved using idxstat in samtools (version 1.9) and placed into count tables for both viral OTUs and MAG clusters [38].

### 2.9. Calculation of Phage-to-Host Ratios

For each viral OTU, the phage-to-host ratio within each sample was calculated by dividing viral fragments per kilobase of transcript per million mapped reads (FPKM) by the sum of the FPKMs of the viral hosts. Phage-to-host ratios were not calculated in cases where none of the hosts received at least 100 mapped reads to avoid falsely-high phage-to-host ratios.

### 2.10. Analysis of Ecological Diversity

For all subsequent analyses, a set of 1290 samples with more than 1,000,000 reads mapped to MAG contigs and viral OTUs was used. Alpha diversity within each stool sample was assessed using Shannon’s diversity index analysis of genome-length-normalized count tables after rarefying at the minimum sample depth using phyloseq R package (version 1.30.0) [39]. This process was repeated 10 times and the average value for each sample was reported. The following linear mixed-effects model was used to assess the statistical significance of the effect of diagnosis factor on the alpha diversity of gut microbial communities using the nlme R package (version 3.1-147) [40]:

$$Shannon^{’}s\;diversity\;index\;\sim \;disease\;status+sex+consent\;age+antibiotics+chemotherapy+\;immunosuppressants+site\;name+\left(1|participant\;ID\right).$$

Significance (*p* value) was determined using the Wald test. Richness was also quantified using the approach described above.

Beta diversity among gut microbial communities was assessed using Bray–Curtis dissimilarity analysis of genome-length-normalized count tables after rarefying using phyloseq R package (version 1.30.0) [39]. The rarefying depth was set at the depth of the smallest sample. The effect of individuality on variance in the gut microbial communities was quantified using PERMANOVA implemented in phyloseq R package’s Adonis function [39,41]. The same method was used to quantify the effect of diagnosis on gut microbiota variance; however, rarefied counts were first averaged for each individual prior to genome-length-normalization. Dissimilarity matrices were visualized in two dimensions using uniform manifold approximation and projection for dimension reduction (UMAP) (version 0.4.2) (n_neighbors = 30, min_dist = 0.3, n_components = 2, metric = braycurtis) [42]. Parallel computations were conducted using GNU parallel (version 20180322) [43].

### 2.11. Analysis of Differential Abundance

Differences in the abundance of gut microorganisms between different diagnosis groups were assessed using the fitZig function in metagenomeSeq R package (version 1.30.0) [44]. First, viral OTUs or MAG clusters that were not present in more than 25% of samples in one of the diagnosis groups were removed. Subsequently, read counts were normalized using the cumulative sum scaling method in metagenomeSeq [44]. A zero-inflated Gaussian mixture model fitted to the normalized read counts was then used to test for differential abundance between the diagnosis groups at a false discovery rate (FDR) cutoff of 0.1 and a log_2_ fold-change cutoff of 1. The FDR was calculated using the Benjamini–Hochberg method. The model used patient ID as a blocking factor along with the following covariates: disease status, sex, consent age (i.e., age of the patient at consent), antibiotics, immunosuppressants, site name, and normalization factor. As advised by Paulson et al. (2013), the normalization factor was set using the median scaling factor [44]. Maximum iteration was set to 100 (default 10). In addition, significant features were restricted to those with an effective sample size greater than or equal to the average as recommended by the authors [44]. To further minimize the possibility of false positives, only those significant features for which the median abundance in the higher diagnosis group was greater than that in the lower diagnosis group were retained. Ten samples belonging to a CD patient (participant ID C3031) were excluded from this analysis because consent age, metadata required in the current zero-inflated Gaussian mixture model, were unavailable.

### 2.12. Data Availability 

Count tables, metadata, and the sequences of MAGs and viral regions can be obtained from the following site: ftp://ftp.genome.jp/pub/db/community/ibd-phage/.

## 3. Results

### 3.1. Taxonomic Classification of Temperate Bacteriophages in the Human Gut

**Figure 1:**
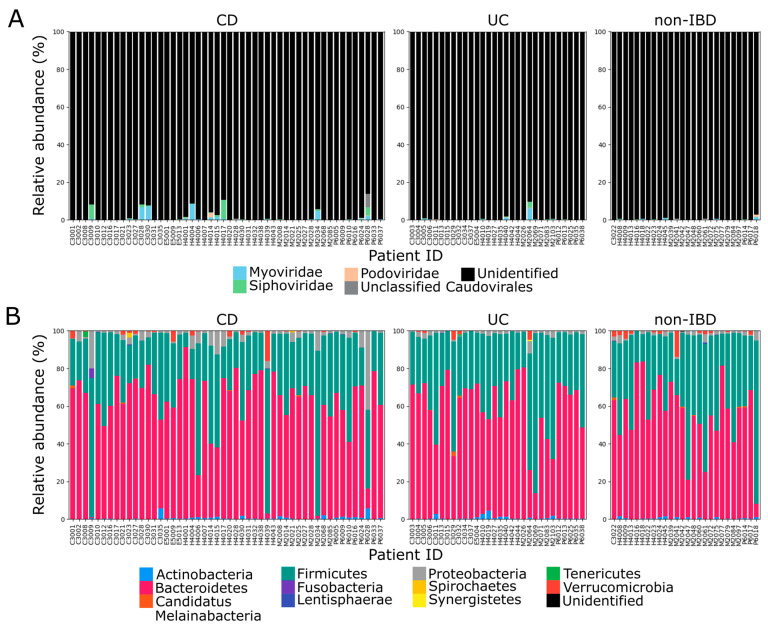
Relative abundance of gut microbes and viruses in inflammatory bowel disease (IBD) and non-IBD patients. Mean relative abundance within each patient is plotted at the family level for temperate bacteriophages (**A**) and at the phylum level for bacteria (**B**). Relative abundance was calculated based on genome length normalized read counts.

A total of 17,536,516 genome fragments were assembled from the 1327 sets of WMGS data generated by the IBDMDB team from stool samples from IBD patients and other patients diagnosed with non-IBD gastrointestinal disorders. To characterize the temperate bacteriophage community in the gut, 50,624 regions predicted to be viral based on the presence of hallmark viral genes were clustered into 17,331 species-rank virus groups based on sequence similarity. Of these viral OTUs, 3843 were associated with putative host sequences and found to encode integrase genes. A total of 1797 viral OTUs with a phage-to-host ratio >10 in at least one sample were conservatively classified as functional (i.e., non-defunct) temperate bacteriophages. The representative viral regions of these viral OTUs ranged in size from 4331 to 313,294 bp, with a median size of 33,609 bp. Only 28 (1.56%) of the temperate bacteriophage OTUs were taxonomically annotated. These belonged to one of the following three *Caudovirales* families: *Myoviridae*, *Podoviridae*, and *Siphoviridae* (Figure 1A).

### 3.2. Infection of a Phylogenetically Wide Range of Human Gut Bacteria by Temperate Bacteriophages

To determine the host distribution of temperate bacteriophages in the human gut, bacterial genomes were reconstructed from metagenome data. By binning contigs based on read coverage and k-mer occurrence, 15,906 MAGs with completion >70% and contamination <10% were reconstructed. These MAGs were then dereplicated into 3133 bacterial MAG clusters based on gANI values. The representative MAGs for each of the MAG clusters ranged in size from 685,598 to 6,988,682 bp, with a median size of 2,635,319 bp. Most (91.3%) of the MAGs were identified as belonging to the phyla *Bacteroidetes* and *Firmicutes*, which are the two most dominant phyla in the human gut (Figure 1B) [45].

**Figure 2:**
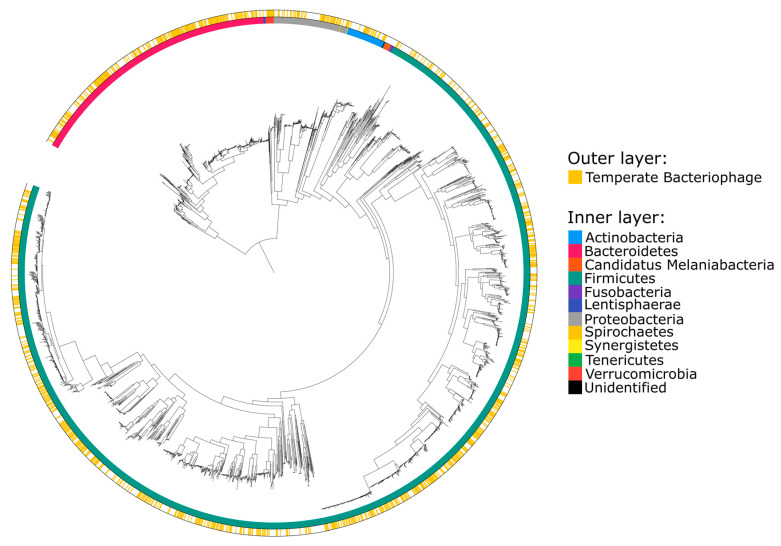
Phylogenetic tree based on a comprehensive collection of reconstructed bacterial genomes. The inner layer colors correspond to the bacterial phyla represented at the tip of each branch. The outer layer indicates bacteria associated with functional temperate bacteriophages.

**Figure 3:**
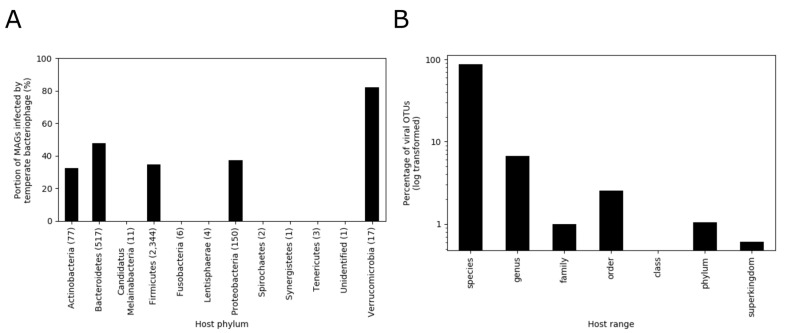
Characteristics of phage-host associations. (**A**) Distribution of temperate bacteriophage host phyla. The numbers following the phylum names indicate the number of metagenome-assembled genomes (MAG) clusters obtained for each phylum. (**B**) Host range of temperate bacteriophages.

A total of 1153 MAG clusters (36.8%) were found to be infected with temperate bacteriophages based on sequence comparisons (Figure 2). Taxonomic classification of the MAG clusters showed that temperate bacteriophages infect a wide range of bacteria distributed across the *Actinobacteria*, *Bacteroidetes*, *Firmicutes*, *Proteobacteria*, and *Verrucomicrobia* phyla (corresponding to 12 classes, 13 orders, 28 families, 63 genera, and 101 species) (Figure 3A, Table S1). To understand the host range of temperate bacteriophages, the last common ancestors of host taxonomies were also calculated (Figure 3B). Based on these results, 94.8% of viral OTUs were found to have a narrow host range at the species and genus levels.

### 3.3. Alpha Diversity of Gut Microbes in IBD and Non-IBD Patients

Dysbiosis is a state of microbiota imbalance characterized by factors such as decreased diversity and altered composition [46]. The gut microbiota from IBD patients has been shown to be dysbiotic [46]. To investigate differences in alpha diversity between IBD patients and patients with non-IBD gastrointestinal disorders, Shannon’s diversity and richness of bacterial and temperate bacteriophage communities were investigated (Figures S1B,C and S2B,C). The same measures were also used to assess the alpha diversity of induced temperate bacteriophage (i.e., temperate bacteriophages in the lytic cycle) communities in each sample. Temperate bacteriophages were conservatively considered to be induced (i.e., active) only when the phage-to-host ratio was >10, based on the threshold utilized in a previous study [35] (Figures S1A and S2A). Consistent with the results from the IBDMDB research group [1], most of the comparisons displayed lower alpha diversity in the IBD patients than in the non-IBD patients, but the comparisons lacked statistical significance (*p* > 0.05 with Holm’s correction).

### 3.4. Variation of Gut Microbial Community Composition among Stool Samples

**Figure 4:**
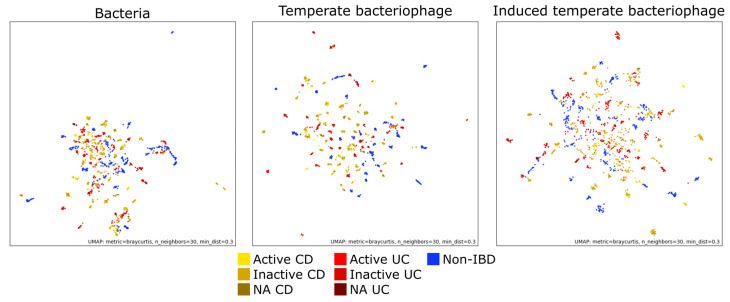
Ordination of gut microbial communities. Dissimilarity among gut microbial communities was assessed using the Bray–Curtis dissimilarity measure and ordinated in two dimensions using uniform manifold approximation and projection for dimension reduction (UMAP). The following samples were used: 87 active Crohn’s disease (CD) samples from 24 patients, 448 inactive CD samples from 48 patients, 45 NA CD samples from 17 patients, 29 active ulcerative colitis (UC) samples from 9 patients, 298 inactive UC samples from 29 patients, 21 NA UC samples from 7 patients, and 362 non-IBD samples from 26 patients. NA CD and NA UC samples refers to those lacking Harvey–Bradshaw index (HBI) and simple clinical colitis activity index (SCCAI) scores, respectively.

To assess variation in microbial community composition among samples, Bray–Curtis dissimilarity between all samples was assessed and results were plotted in two dimensions using UMAP (Figure 4). Consistent with previous reports, the temperate bacteriophage and bacterial community compositions were highly specific to each individual (effect size = 71.2% and 69.7% for temperate bacteriophage and bacterial communities, respectively; *p* < 0.0001, PERMANOVA test) [1,47]. The effect of individuality on the composition of induced temperate bacteriophage communities could not be estimated because of a lack of induced temperate bacteriophages in many samples. The effect of disease status on microbial community composition was also examined but differences were not significant (*p* > 0.05, PERMANOVA test).

### 3.5. Differential Abundance of Temperate Bacteriophages and Their Hosts

**Figure 5:**
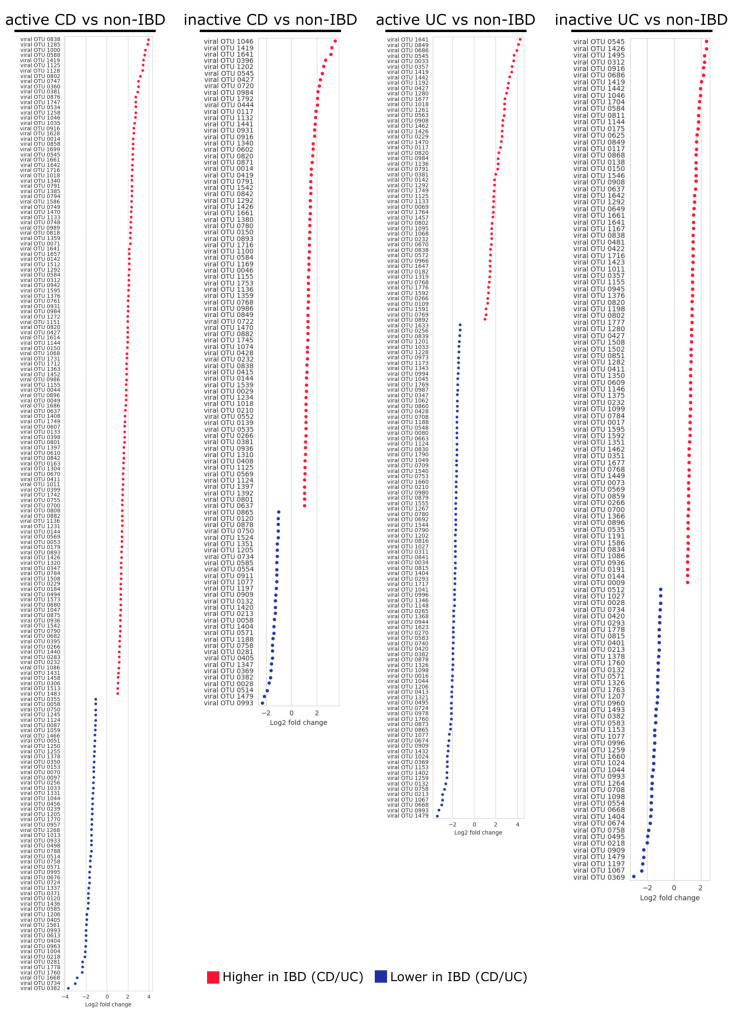
Log_2_ fold-change values for temperate bacteriophages displaying statistically significant differences in abundance between IBD and non-IBD patients (FDR < 0.1). Positive fold-change values indicate a higher abundance in IBD patients, whereas a negative fold-change indicates higher abundance in non-IBD patients. The following samples were used: 87 active CD samples from 24 patients, 448 inactive CD samples from 48 patients, 29 active UC samples from 9 patients, 298 inactive UC samples from 29 patients, and 362 non-IBD samples from 26 patients.

**Figure 6:**
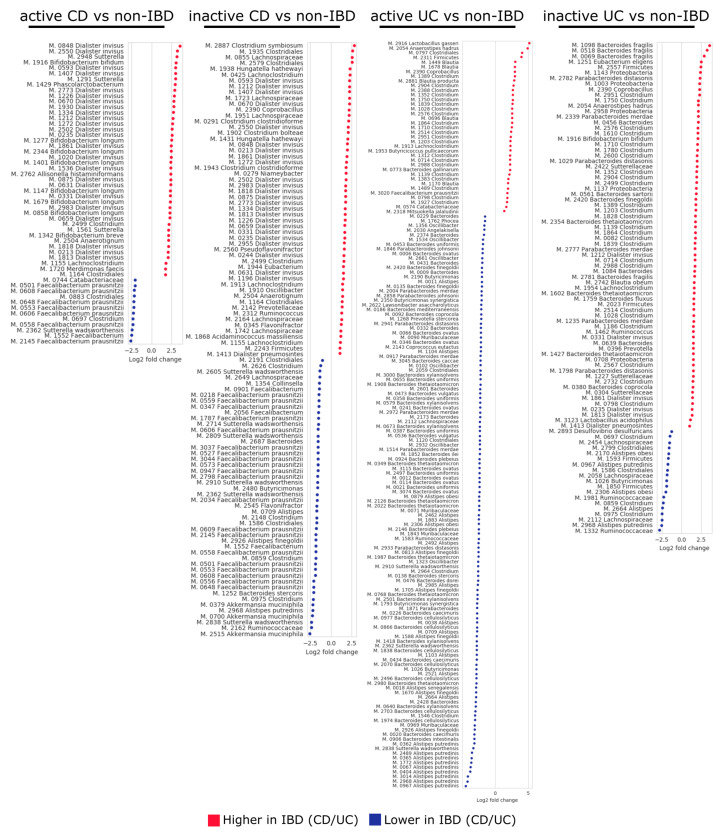
Log_2_ fold-change values for bacteria displaying statistically significant differences in abundance between IBD and non-IBD patients (FDR < 0.1). Positive fold-change indicates a higher abundance in IBD patients, whereas a negative fold-change indicates higher abundance in non-IBD patients. MAG cluster is abbreviated to “M.” The following samples were used: 87 active CD samples from 24 patients, 448 inactive CD samples from 48 patients, 29 active UC samples from 9 patients, 298 inactive UC samples from 29 patients, and 362 non-IBD samples from 26 patients.

To gain a better understanding of the role played by the microbial community in IBD at a finer scale, differences in the abundances of the temperate bacteriophages and bacteria between the IBD and non-IBD patients were assessed at the level of viral OTUs and MAG clusters, respectively (Figure 5 and Figure 6). In comparisons between CD and non-IBD patients, 182 and 105 viral OTUs were found to be differentially abundant between active CD and non-IBD patients and between inactive CD and non-IBD patients, respectively (FDR < 0.1) (Tables S2 and S4). Additionally, 54 and 102 MAG clusters were shown to be differentially abundant between active CD and non-IBD patients and between inactive CD and non-IBD patients, respectively (FDR < 0.1) (Tables S3 and S5). Over-represented MAG clusters in CD patient samples predominantly consisted of *Dialister invisus* (active CD: 24 MAG clusters, inactive CD: 24 MAG clusters), whereas under-represented MAG clusters in these patients were largely attributed to *Faecalibacterium prausnitzii* (active CD: 7 MAG clusters, inactive CD: 20 MAG clusters). Of note, similar differences in abundance (i.e., the same trend of over-/under-representation) were not observed for bacterial hosts of the differentially-abundant viral OTUs (except in two cases) in any of the comparisons (active CD vs non-IBD and inactive CD vs non-IBD).

Comparisons between UC and non-IBD patients yielded 145 and 126 viral OTUs that were differentially abundant between active UC and non-IBD patients and between inactive UC and non-IBD patients, respectively (FDR < 0.1) (Tables S6 and S8). In addition, 160 and 89 MAG clusters were differentially abundant between active UC and non-IBD patients and between inactive UC and non-IBD patients, respectively (FDR < 0.1) (Tables S7 and S9). In the active UC vs. non-IBD comparison, notable differences included an increased abundance of *Clostridium* spp. (21 MAG clusters) and decreased abundance of *Alistipes* spp. (28 MAG clusters) and *Bacteroides* spp. (58 MAG clusters) in the active UC patients compared with the non-IBD patients. The inactive UC vs. non-IBD comparison revealed an increased abundance of *Bacteroides* spp. (14 MAG clusters) and *Clostridium* spp. (25 MAG clusters) in the inactive UC patients compared with the non-IBD patients. As observed in the comparisons between CD and non-IBD patients, differences in the abundance of host MAG clusters were not identified for the majority of the differentially-abundant viral OTUs. With the exception of 49 differentially-abundant viral OTUs identified in the active UC vs. non-IBD comparison and 15 differentially-abundant viral OTUs identified in the inactive UC vs. non-IBD comparison, most of the differences in viral OTU abundance were not reflected in host abundance. Of note, viral OTUs 0791, 0838, 0802, and 1592 were over-represented in active UC patients, whereas their hosts, MAG clusters 0358, 0655, 0655, and 2980, respectively, were under-represented in the same patients. Taxonomical classification analysis identified MAG clusters 0358 and 0655 as *Bacteroides uniformis* and MAG cluster 2980 as *Bacteroides thetaiotaomicron*.

## 4. Discussion

In the current study, we characterized the ecological structure of the temperate bacteriophage community in the guts of IBD-affected individuals by identification of bacterial hosts. Previous studies have reported that the human gut viral community largely consists of unclassified members [3,8,48]. In line with these observations, we were only able to taxonomically assign 28 (1.56%) of the viral OTUs corresponding to the temperate bacteriophages that we identified in the current study. To address this problem, we characterized the temperate bacteriophage community by host assignment based on sequence comparisons between reconstructed microbial and viral genomes. Our findings revealed that a wide range of bacteria across the *Actinobacteria*, *Bacteroidetes*, *Firmicutes*, *Proteobacteria*, and *Verrucomicrobia* phyla are infected with functional temperate bacteriophage in the human gut (Figure 2 and Figure 3A). In addition, the majority (94.8%) of the temperate bacteriophage OTUs were found to possess a narrow host range at the level of species or genus. This finding is similar to the results from a previous study showing that 99% of the phage OTUs from various environments possess a narrow host range at the species or genus level [34].

Analysis of beta diversity in gut microbial communities revealed that community composition was highly specific to each individual, and was not associated with disease status (Figure 4). This result is consistent with a previous report by the IBDMDB research team that suggested that individuality is the most important factor in explaining variance in gut microbial community structure [1]. In addition, another study on gut bacteriophage communities found that community composition was specific to each individual [47]. In the current study, we also observed the same trend for the temperate bacteriophage community in IBD-affected gut samples.

Among the CD vs. non-IBD comparisons (i.e., active CD vs. non-IBD and inactive CD vs. non-IBD), with the exception of two viral OTUs, differential abundance was not observed among the bacterial hosts of viral OTUs that were differentially abundant (Figure 5 and Figure 6). For UC and non-IBD patients, the hosts of 96 (66.2%) and 111 (88.1%) differentially-abundant viral OTUs did not show the same trend in abundance in the active UC vs. non-IBD and inactive UC vs. non-IBD comparisons, respectively. This suggests that the differential abundance displayed by these temperate bacteriophages is not merely a reflection of shifts in host abundance. However, techniques such as the single-cell viral tagging used by Džunková et al. will be needed in order to validate the current results [49].

In the active UC vs. non-IBD comparison, four temperate bacteriophages (viral OTUs 0791, 0838, 0802, and 1592) that were over-represented in active UC patients were accompanied by under-representation of their hosts (*B. uniformis*: MAG clusters 0358 and 0655, *B. thetaiotaomicron*: MAG cluster 2980). Interestingly, both *B. uniformis* and *B. thetaiotaomicron* have been experimentally proven to be beneficial to gut homeostasis [50,51,52]. *B. uniformis* isolated from a healthy fecal donor decreased the expression of pro-inflammatory cytokine interleukin-8 in lipopolysaccharide-induced human colorectal adenocarcinoma cells [50]. In another study, *B. thetaiotaomicron* significantly increased the concentration of interleukin-6 (IL-6) secreted by intraepithelial lymphocytes in the mouse colon, whereas IL-6 deficiency significantly decreased the integrity of the epithelial barrier [51]. Furthermore, administration of *B. thetaiotaomicron* was found to ameliorate colon damage in IBD model mice [52]. Therefore, increased lysis of these beneficial bacteria by temperate bacteriophages may contribute to disease pathogenicity in patients with active UC.

In contrast to our results, a study conducted by the IBDMDB research group reported no differences in microbial abundance between IBD and non-IBD patients [1]. We suspect that this is largely the result of differences in how microbial abundance was assessed. In the current study, microbial abundance was assessed by read mapping to a collection of genome fragments reconstructed from the actual gut microbiota under study. In the previous study, microbial abundance was assessed via read mapping to clade-specific gene markers using MetaPhlAn2 [53]. Although both methods have pros and cons, the current approach was selected for the current study because a large proportion of temperate bacteriophages are yet to be characterized in reference databases.

The current study only explored ecological traits (i.e., alpha diversity, beta diversity, and differential abundances) in temperate bacteriophage communities; thus, no assessments of the functional traits of temperate bacteriophages were conducted. The reason for limiting the design of our study in this way is related to the difficulty involved in recovering full-length temperate bacteriophage genomes from whole metagenome sequence data. Future developments in the metagenome contig binning technique for the viral genomes currently un-represented in reference databases may promote deeper understanding of the relationships between gene functional traits in temperate bacteriophages and IBD. We also acknowledge that our study is limited by the modest number of patients and lack of healthy subjects. Future studies on a larger population of patients and inclusion of healthy subjects will be needed for further confirmation of our results.

In conclusion, our results support growing evidence that the gut temperate bacteriophage community does indeed have a relationship with the pathogenesis of IBD. Notably, when compared with non-IBD patients, the temperate bacteriophages infecting two species that were experimentally shown to be beneficial to gut homeostasis, *B. uniformis* and *B. thetaiotaomicron*, were over-represented in patients with active UC. Worth mentioning is that, our study has reconstructed a large collection of functional temperate bacteriophage and bacterial genomes that may be of benefit to future studies. Further understanding of the role played by temperate bacteriophages in the human gut microbiota has potential to lead to the development of new phage therapy for IBD.

## Supplementary Materials

The following are available online at https://www.mdpi.com/2076-2607/8/11/1663/s1: Figure S1: Comparisons of Shannon’s diversity index values for bacteria and temperate bacteriophages between IBD and non-IBD patients; Figure S2: Comparisons of bacterial and temperate bacteriophage community richness between IBD and non-IBD patients; Table S1. Temperate bacteriophages and their assigned hosts; Table S2: Differentially-abundant viral OTUs between active CD and non-IBD patients with FDR values < 1.0; Table S3: Differentially-abundant MAG clusters between active CD and non-IBD patients with FDR values < 1.0; Table S4: Differentially-abundant viral OTUs between inactive CD and non-IBD patients with FDR values < 1.0; Table S5: Differentially-abundant MAG clusters between inactive CD and non-IBD patients with FDR values < 1.0; Table S6: Differentially-abundant viral OTUs between active UC and non-IBD patients with FDR values < 1.0; Table S7: Differentially-abundant MAG clusters between active UC and non-IBD patients with FDR values < 1.0; Table S8: Differentially-abundant viral OTUs between inactive UC and non-IBD patients with FDR values < 1.0; Table S9: Differentially-abundant MAG clusters between inactive UC and non-IBD patients with FDR values < 1.0.
