# Supplementary figures and images for: Ecological Structuring of Temperate Bacteriophages in the Inflammatory Bowel Disease-Affected Gut
Source: Microorganisms. 2020 Oct 27;8(11):1663. doi: 10.3390/microorganisms8111663 (PMC7692956; doi:10.3390/microorganisms8111663)

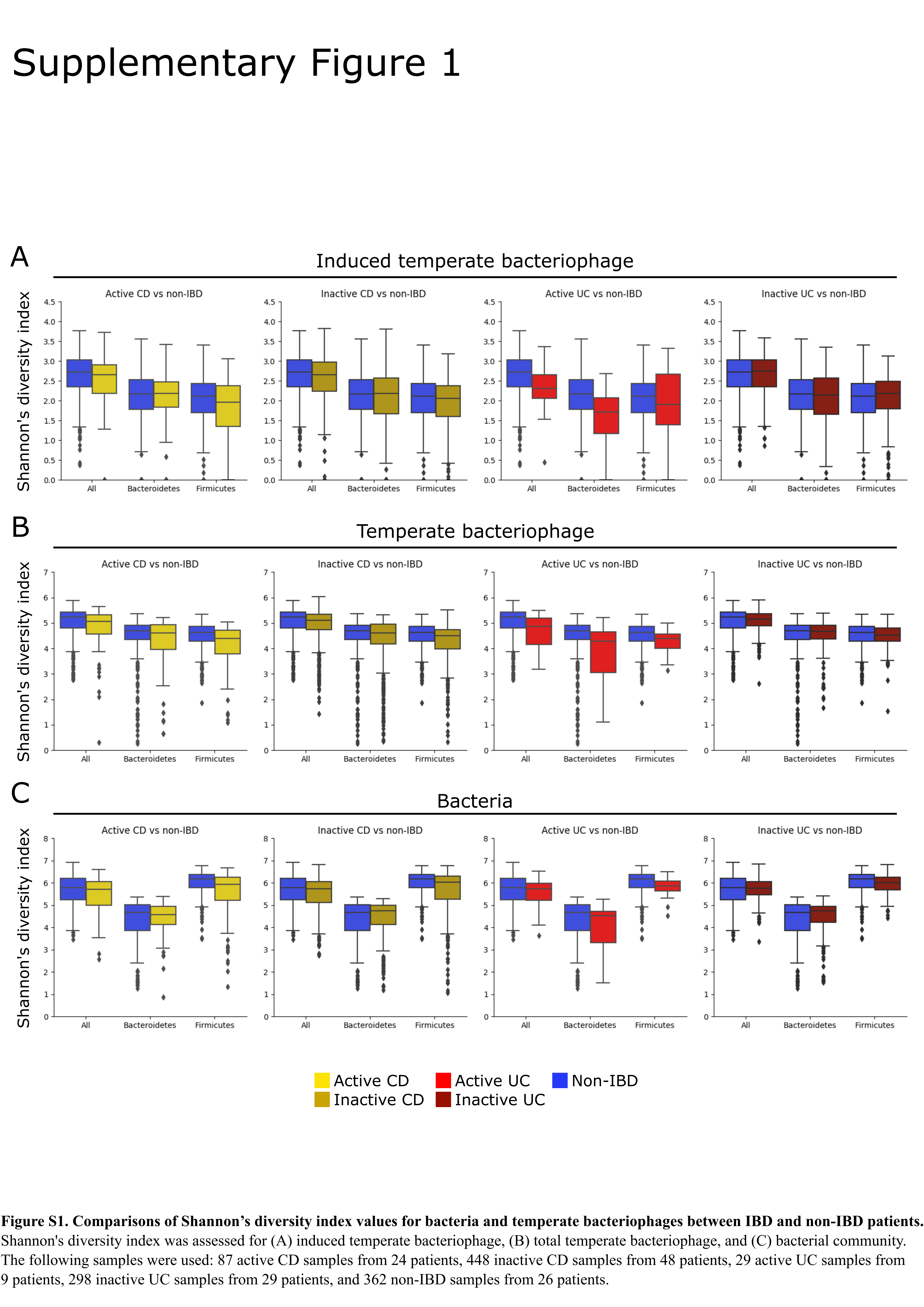

Supplement: Supplementary file 1 [file microorganisms-08-01663-s001.zip › FigS1 Shannon's diversity index value.tiff]

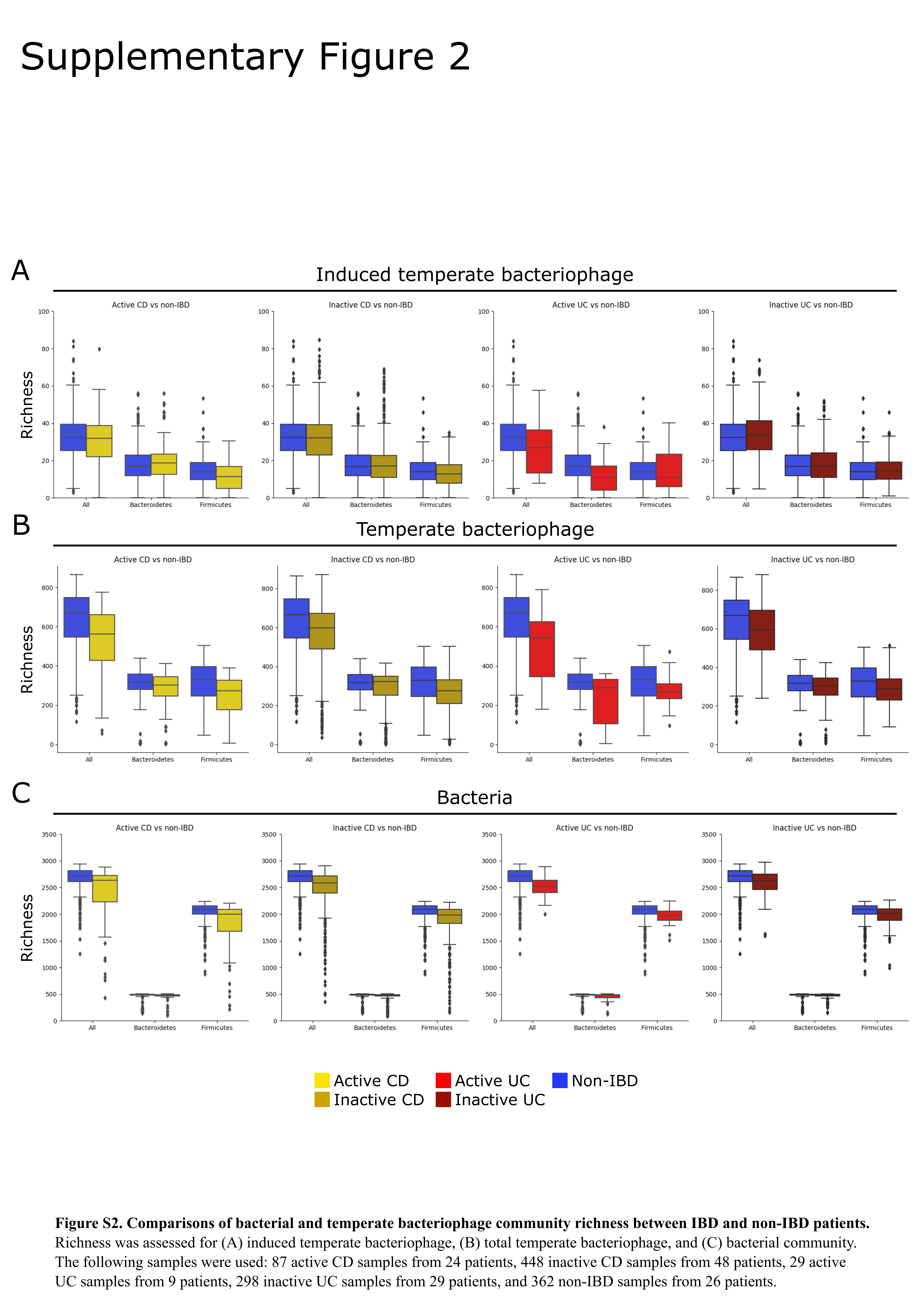

Supplement: Supplementary file 1 [file microorganisms-08-01663-s001.zip › FigS2 Richness.tiff]
